# A new species of *Theridion* Walckenaer, 1805 (Araneae, Theridiidae) from Ganjiangyuan National Nature Reserve, southern China

**DOI:** 10.3897/BDJ.14.e182063

**Published:** 2026-01-07

**Authors:** Mingshan Tan, Kuankuan Xu, Guchun Zhou

**Affiliations:** 1 School of Life Sciences, National Navel Orange Engineering Research Center, Gannan Normal University, Ganzhou, Jiangxi 341000, China School of Life Sciences, National Navel Orange Engineering Research Center, Gannan Normal University Ganzhou, Jiangxi 341000 China; 2 Forestry Bureau of Ruijin City, Ganzhou, Jiangxi 341000, China Forestry Bureau of Ruijin City Ganzhou, Jiangxi 341000 China

**Keywords:** Asia, cobweb spiders, distribution, morphology, taxonomy

## Abstract

**Background:**

The genus *Theridion* Walckenaer, 1805 comprises 577 species, distributed all over the world and it is common in habitat species, such as grasses, barks and deciduous layers.

**New information:**

A new cobweb species, *Theridion ganjiangyuan* Zhou, sp. nov., is described from the Ganjiangyuan National Nature Reserve, in Jiangxi Province, China. A detailed description, diagnosis, illustrations and a distribution map of the new species are provided.

## Introduction

The comb-footed spider family Theridiidae Sundevall, 1833 is the fourth largest family of spiders in the world, with 136 genera and 2622 species (World Spider Catalog 2025). *Theridion* Walckenaer, 1805 is the most speciose genus within the family Theridiidae, currently comprising 577 recorded species. However, it is also the most taxonomically confused genus regarding species composition. The main characteristics of the genus *Theridion* include: (1) the absence of a colulus at the edge of the spinnerets on the ventral side of the abdomen; (2) the male pedipalps exhibit a distinct median apophysis (MA) and theridiid tegular apophysis (TTA), with clearly defined structures for insertion and guidance; and (3) the female atrium is distinctly defined, possessing two copulatory opening (Zhu 1998). By clarifying these characteristics to identify the fundamental features of the genus, it can be determined that many species may be removed from this genus or established as new genera. It is hoped that the revision of this genus can be expedited. In China, 61 species of this genus have been recorded, primarily distributed in the southern regions.

The Ganjiangyuan National Nature Reserve is located at the confluence of Shicheng County and Ruijin City, spanning a geographical range from 25°56'N to 26°7'N and from 116°15'E to 116°29'E, with its highest peak reaching an elevation of 1389.9 metres (Zhou et al. 2023). The climate of the Reserve is classified as a temperate humid subtropical monsoon climate, which is characterised by abundant rainfall, brief periods of extreme heat and cold and an extended frost-free season. Notable climatic features include prevailing winter and summer monsoons, concentrated precipitation during the spring and summer months and frequent heavy rainfall events. These favourable thermal conditions, along with ample rainfall and concentrated precipitation patterns, contribute to the climatic characteristics that promote soil development within the Reserve, while simultaneously fostering a conducive environment for the area's biodiversity (Zhou et al. 2023, Wang et al. 2024).

## Materials and methods

Specimens were collected using handpicking and sweeping methods, then preserved in 80% ethanol. The female genitalia were dissected and cleared in a trypsin enzyme solution prior to examination and photography. The male pedipalpus was soaked in a 50% lactic acid solution for two hours, then observed and photographed. Specimens were examined and measured using a Leica MZ6 stereomicroscope. Photographs were taken with a Kuy Nice CCD mounted on an Olympus BX41 and processed using Helicon Focus software (version 3.10, free) (Khmelik et al. 2005). Maps were created using ArcGis 10.2 and subsequently modified with Adobe Photoshop 2021 Extended. Leg measurements are provided in the following order: total length (femur, patella, tibia, metatarsus, tarsus), with all measurements expressed in millimetres (mm). The specimens are deposited in the Taxidermy Museum of Gannan Normal University, Ganzhou City, China (GNNU).

Terminology and taxonomic descriptions follow Gao and Li (2014) and Zhou et al. (2024). Abbreviations: **ALE** = anterior lateral eye; **AME** = anterior median eye; **C** = conductor; **CD** = copulatory duct; **CO** = copulatory orifice; **E** = embolus; **EB** = embolic base; **FD** = fertilisation duct; **MA** = median apophysis; **MOA** = median ocular area; **PLE** = posterior lateral eye; **PME** = posterior median eye; **S** = spermatheca; **ST** = subtegulum, **T** = tegulum, **TTA** = theridiid tegular apophysis.

## Taxon treatments

### Theridion ganjiangyuan

Zhou sp. nov.

1F5FBF30-505D-5FCB-BC7A-526B09D97C56

80933033-F1BC-46F5-B258-5AC0A31C60B9

#### Materials

- **Type status:** Holotype. **Occurrence:** recordedBy: Zhou G.C.; individualCount: 1; sex: male; lifeStage: adult; occurrenceID: 8BC04749-60E6-5401-95AF-3F4E47EE613D; **Taxon:** kingdom: Animalia; phylum: Arthropoda; class: Arachnida; order: Araneae; family:  Theridiidae; genus: Theridion; **Location:** country: China; stateProvince: Jiangxi Province; county: Ruijin City; locality: Ganjiangyuan National Nature Reserve, Ridong Township, Ruijin City, Ganzhou City; verbatimElevation: 349; verbatimLatitude: 25°57'19.512''N; verbatimLongitude: 116°16'18.437''E; **Event:** samplingProtocol: by hand; year: 2024; month: 7; day: 19; **Record Level:** institutionCode: JXGJY-24-47-04
- **Type status:** Paratype. **Occurrence:** recordedBy: Zhou G.C.; individualCount: 1; sex: male; lifeStage: adult; occurrenceID: 655B8922-728B-50FB-8CA3-3F324889FE57; **Taxon:** kingdom: Animalia; phylum: Arthropoda; class: Arachnida; order: Araneae; family:  Theridiidae; genus: Theridion; **Location:** country: China; stateProvince: Jiangxi Province; county: Ruijin City; locality: Ganjiangyuan National Nature Reserve, Ridong Township, Ruijin City, Ganzhou City; verbatimElevation: 349; verbatimLatitude: 25°57'19.512''N; verbatimLongitude: 116°16'18.437''E; **Event:** samplingProtocol: by hand; year: 2024; month: 7; day: 19; **Record Level:** institutionCode: JXGJY-24-47-05
- **Type status:** Paratype. **Occurrence:** recordedBy: Zhou G.C.; individualCount: 1; sex: female; lifeStage: adult; occurrenceID: C8B9FABD-F3A7-5473-8FF8-A7E845C6A6AD; **Taxon:** kingdom: Animalia; phylum: Arthropoda; class: Arachnida; order: Araneae; family:  Theridiidae; genus: Theridion; **Location:** country: China; stateProvince: Jiangxi Province; county: Ruijin City; locality: Ganjiangyuan National Nature Reserve, Ridong Township, Ruijin City, Ganzhou City; verbatimElevation: 349; verbatimLatitude: 25°57'19.512''N; verbatimLongitude: 116°16'18.437''E; **Event:** samplingProtocol: by hand; year: 2024; month: 7; day: 19; **Record Level:** institutionCode: JXGJY-24-47-06

#### Description

**Figure 1.**
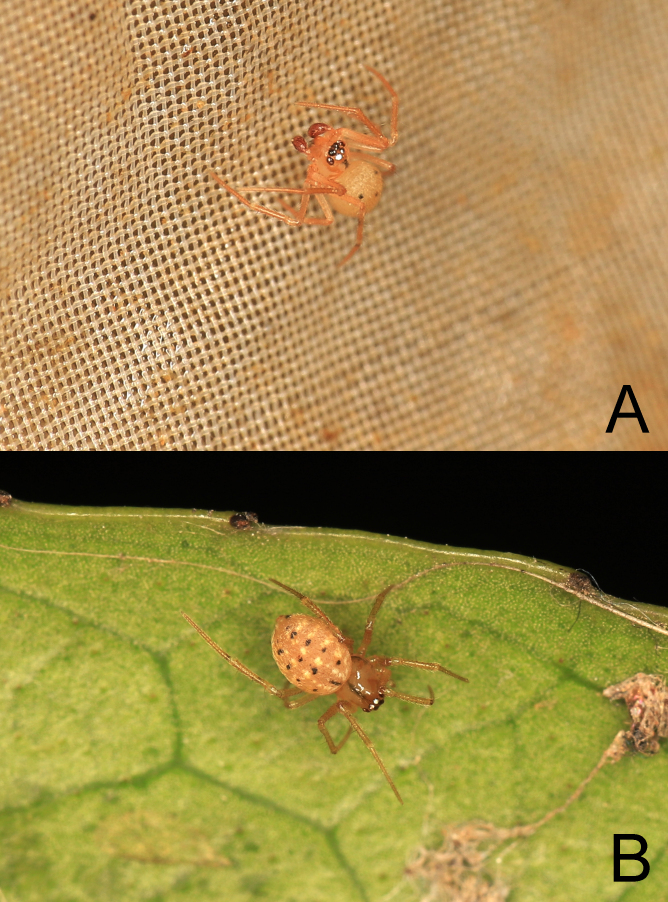
Living photos of *Theridion ganjiangyuan* Zhou, sp. nov.: **A** male (holotype); **B** female (paratype).

**Figure 2.**
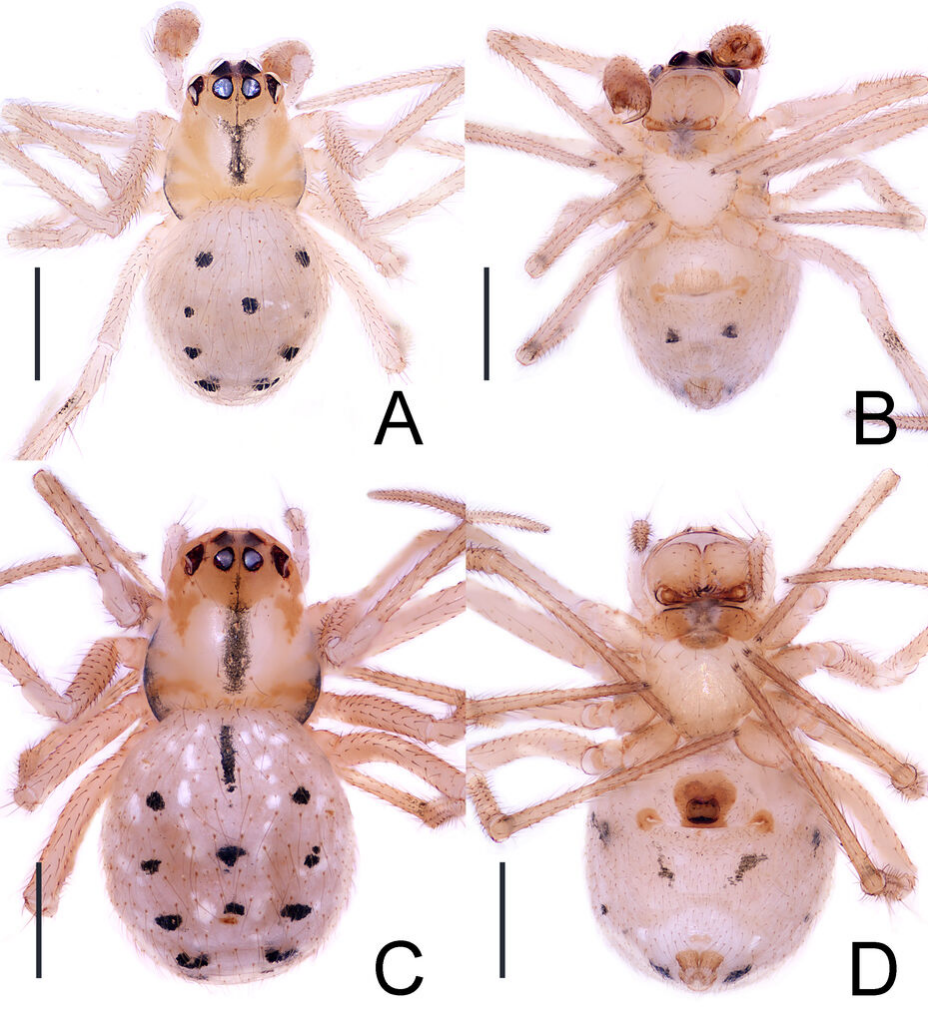
*Theridion ganjiangyuan* Zhou, sp. nov., habitus, **A-B** male (holotype); **C-D** female (paratype). **A, C** dorsal view; **B, D** ventral view. Scale bars: 0.5 mm (A-D).

**Male** (holotype, JXGJY-24-47-04): Habitus as in Fig. 1A and Fig. 2A and B. Total length 1.69. Prosoma 0.76 long, 0.63 wide, exhibiting deep yellow radioactive rays in the dorsal view (Fig. 2A). Sternum 0.16 long, 0.17 wide, pale yellow, grey-black spots along the edges. Opisthosoma 0.93 long, 0.83 wide, covered in fine hairs and adorned with more than ten round black spots in the dorsal view (Fig. 2A), as well as two black spots near triangular and without colulus in ventral view (Fig. 2B). Eye measurements: AME 0.09, ALE 0.07, PME 0.09, PLE 0.09, AME–AME 0.07, AME–ALE 0.03, PME–PME 0.05, PME–PLE 0.06; the edge of the eye is black. MOA: 0.19 long; 0.22 anterior width, 0.21 posterior width. Clypeus height 0.11. Chelicerae: multiple long and brown spines, 2 prolateral teeth, 3 retrolateral teeth. Leg measurements: I 2.76 (0.88, 0.25, 0.59, 0.63, 0.41); II 2.43 (0.83, 0.22, 0.55, 0.49, 0.34); III 1.88 (0.53, 0.21, 0.41, 0.42, 0.31); IV 2.48 (0.82, 0.21, 0.51, 0.61, 0.33). Leg formula: 1423.

**Figure 3.**
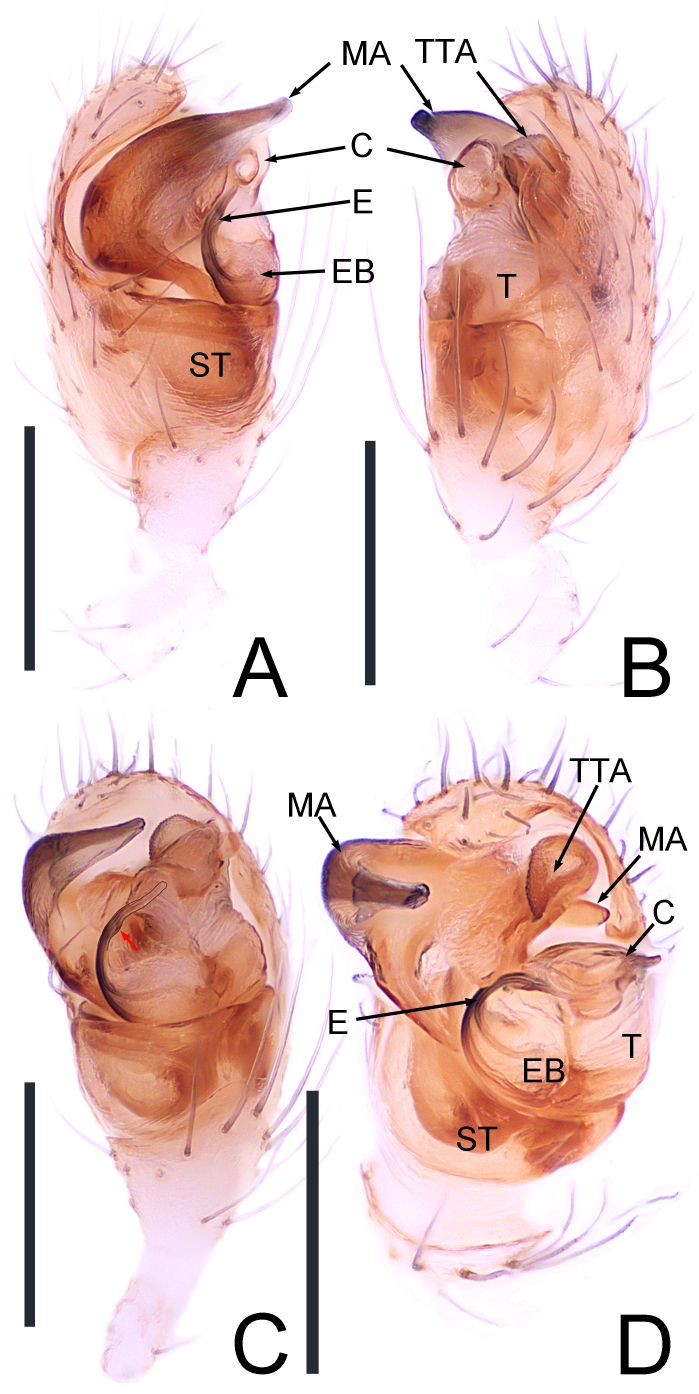
*Theridion ganjiangyuan* Zhou, sp. nov.: holotype male. **A** Pedipalpus, prolateral view; **B** Pedipalpus,, retrolateral view; **C** Pedipalpus, ventral view; **D** Pedipalpus, ventral view. Abbreviations: C = conductor, E = embolus, EB = embolic base, MA = median apophysis, ST = subtegulum, T = tegulum, TTA = theridiid tegular apophysis. Scale bars: 0.2 mm (A-D).

**Pedipalpus** (Fig. 3A-D). Median apophysis (MA) horn-like, pointing to the 2 o'clock direction (Fig. 3C); theridiid tegular apophysis (TTA) upper edge arranged in row and features small particle protrusions on its surface (Fig. 3B); embolic base (EB) wider, pointing to the left, exhibiting a beak shape (Fig. 3C); embolus (E) contains a distinct seminal sperm duct in the middle, appearing black with small protrusions (red arrow) along the middle (Fig. 3C); the inner base of the conductor (C) has a cavity positioned centrally (Fig. 3A), which the upper end is transversely cracked (Fig. 3D).

**Figure 4.**
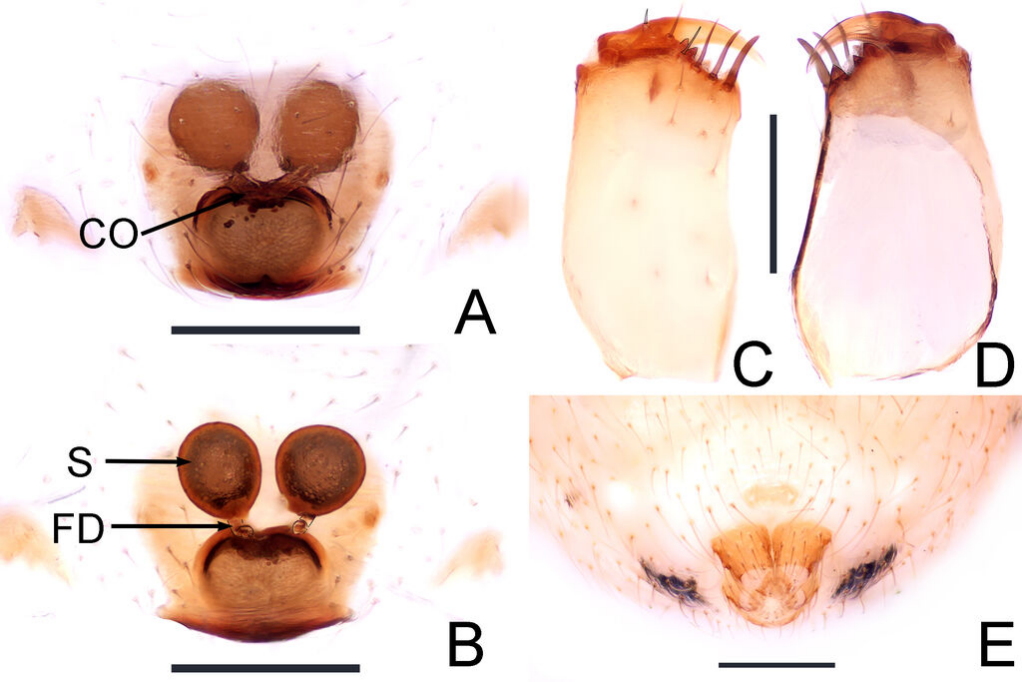
*Theridion ganjiangyuan* Zhou, sp. nov., paratype female. **A** Epigynum, ventral view; **B** Vulva, dorsal view; **C** Chelicera, anterior view; **D** Chelicera, posterior view; **E** Spinnerets, ventral view. Abbreviations: CO = copulatory opening, FD = fertilisation duct, S = spermatheca. Scale bars: 0.2 mm (A-E).

**Female** (paratype, JXGJY-24-47-06): Habitus as in Fig. 1B and Fig. 2C and D. Total length 2.11. Prosoma 0.89 long, 0.76 wide, characterised by a dark yellow colouration on the head region, which lacks a middle fossa on the carapace and features grey-black longitudinal strips (Fig. 2C). Sternum 0.46 long, 0.53 wide, pale yellow colour with grey-black spots along the edges. Opisthosoma 1.22 long, 1.11 wide, with a labium (Fig. 2D). The labium is yellowish-brown (Fig. 2D). Eye measurements: AME 0.08, ALE 0.08, PME 0.09, PLE 0.09, AME–AME 0.09, AME–ALE 0.04, PME–PME 0.03, PME–PLE 0.09; the edge of the eye is black. MOA: 0.19 long; 0.22 front width, 0.21 back width. Clypeus height 0.15. Chelicerae: multiple long and brown spines, 2 prolateral teeth, 3 retrolateral teeth (Fig. 4C and D). Leg measurements: I 2.91 (1.01, 0.26, 0.61, 0.61, 0.42); II 2.61 (0.89, 0.24, 0.57, 0.64, 0.37); III 2.25 (0.74, 0.22, 0.49, 0.49, 0.31), IV 3.15 (1.02, 0.31, 0.69, 0.71, 0.41). Leg formula: 4123. The spinnerets of the abdomen, viewed ventraly, show no colulus (Fig. 4E).

**Epigynum** (Fig. 4A and B). Atrium (A) wider and nearly rounded and the posterior edge slightly raised. Copulatory opening (CO) located at the middle position of the anterior atrium, with the two openings neighbouring and embolised (Fig. 4A); copulatory duct (CD) shorter, leading directly to the spermatheca (S), which is nearly spherical; fertilisation duct (FD) horn-shaped, with the ends bent to both sides (Fig. 4B).

#### Diagnosis

The newly-identified species exhibits a resemblance to *Theridion submirabile* Zhu & Song, 1993 (Zhu 1998: fig. 81A-F; Yang 2019: figs. 105 and 106) due to the similarity in abdominal patterns observed in both male and female specimens. However, the male can be distinguished by the following characteristics: (1) the median apophysis (MA) points to the 2 o'clock direction (Fig. 3C) in contrast to the MA of *T. submirabile* which points to the 1 o'clock direction (Yang 2019: fig. 105b); (2) the theridiid tegular apophysis (TTA) has its upper edge arranged in a row (Fig. 3B), whereas the TTA of *T. submirabile* features micro protrusions (Yang 2019: fig. 105d); (3) the embolus (E) directs towards the upper prolateral, as opposed to the embolus of *T. submirabile*, which initially points horizontally to the right before curving upwards (Yang 2019: fig. 105b). In terms of female specimens, distinguishing features include: (1) the atrium (A) is wider and nearly round compared to the less distinct A of *T. submirabile* (Yang 2019: fig. 106b); (2) the copulatory opening (CO) is centrally located and very close together (Fig. 4A) unlike the CO of *T. submirabile*, which is situated on both sides of the centre (Yang 2019: fig. 106b); (3) the length of the copulatory duct (CD) and the width of the copulatory opening (CO) have many differences and shorter (Fig. 4B), in contrast to the longer and more pronounced CD observed in *T. submirabile* (Yang 2019: fig. 106c).

#### Etymology

The specific name refers to the type locality, noun in apposition.

#### Distribution

**Figure 5.**
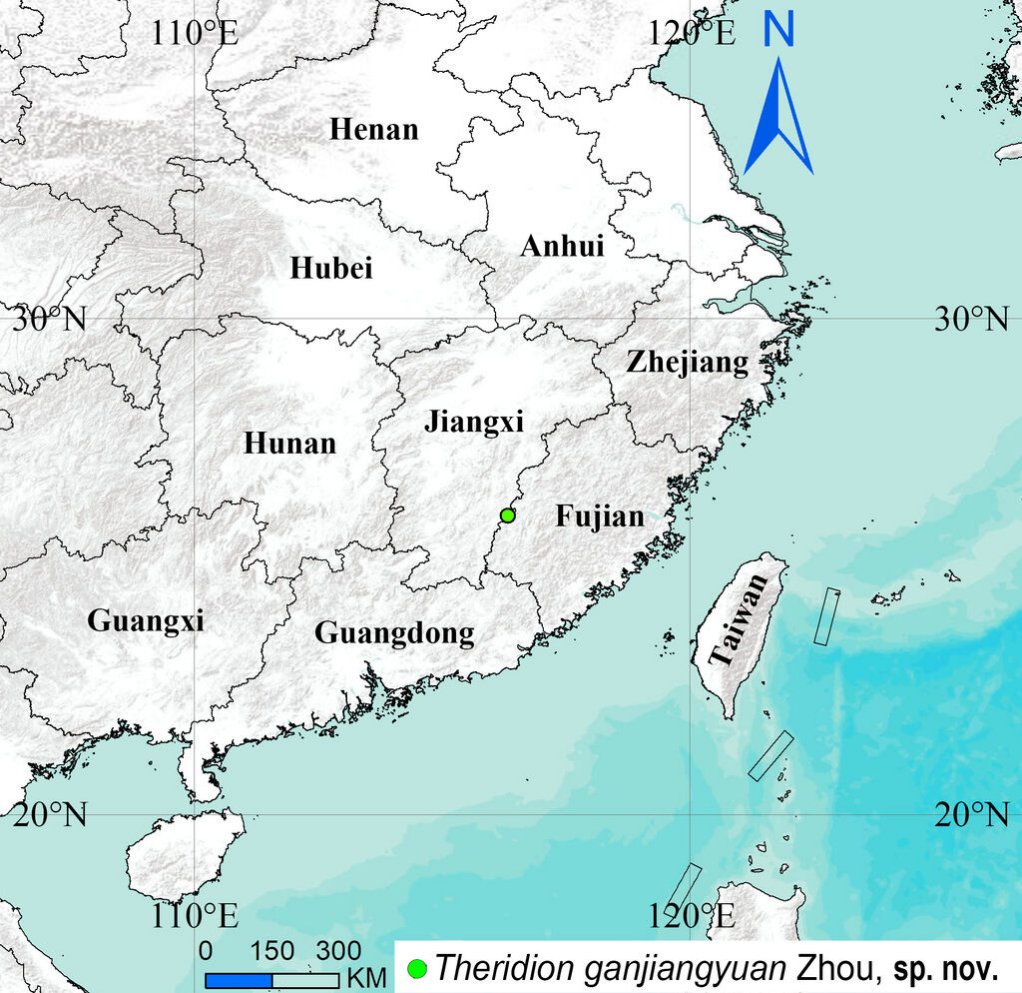
Distribution record of *Theridion ganjiangyuan* Zhou, sp. nov. from Jiangxi Province, China.

Known only from the type locality in Jiangxi Province, China (Fig. 5).

## Discussion

*Theridion submirabile* Zhu & Song, 1993, is primarily distributed in the Fujian, Hainan, Hunan, Chongqing and Hubei Provinces of China (Zhu 1998, Yang 2019), whereas only male specimens have been recorded in South Korea (Namkung 2003). It differs distinctly from *T. s.* in the external morphology of male spiders and the structural characteristics of the male palps. The differences are as follows: (1) The near-circular spots on the dorsal abdomen are uniform in size and evenly distributed across the back, whereas *T. s.* in China exhibits countable black spots concentrated in the middle of the dorsal abdomen and along both sides; (2) The median eminence of the male palps is small in *T. s.* in Korea, but large in *T. s.* in China. Therefore, the species identified as *T. s.* in Korea was misidentified and requires re-examination. Both the new species *T. ganjiangyuan* and *T. s.* lack molecular data for classification and identification. Consequently, it is necessary to determine their genetic relationship through DNA analysis and to establish a new genus separate from *Theridion* as a new combination. The basis for this includes: (1) the diameter of each eye in the new combination is relatively larger than that of common *Theridion* species; (2) the copulatory cavity lacks a septum and is concave outwards. In the future, the new genera could be established as more evidence of new species becomes available.

## Supplementary Material

XML Treatment for Theridion ganjiangyuan
